# Physical education and student well-being: Promoting health and fitness in schools

**DOI:** 10.1371/journal.pone.0296817

**Published:** 2024-01-25

**Authors:** Hui Sun, Cheng-Run Du, Zhi-Feng Wei

**Affiliations:** 1 Business Development Department, Beijing Open University, Beijing, China; 2 School of Sports Management and Communication, Capital University of Physical Education and Sports, Beijing, China; 3 Department of Sports and Art Teaching, Beijing Technology and Business University, Beijing, China; 4 China Volleyball Collage, Beijing Sport University, Beijing, China; University of Tartu, ESTONIA

## Abstract

The school students are facing mental health issues, and their performance is not improving in China. Health education policies are not implemented at the school level in China. However, scholars focus on college students’ health education, but the school student is neglected. The research’s primary objective is to answer the question: What is the impact of health education on the psychological well-being of school students? A sample of 549 10^th^ grade students is collected from China’s public and private sector institutes. The partial least square–structural equation modelling (PLS-SEM) is employed to analyze the data. The outcomes highlighted that the impact of health education is significant on the psychological well-being of school students in China. Furthermore, the study introduced that the moderating role of sustainable health exercise and sports participation is critical as it positively influences the relationship between health education and psychological wellbeing. This research improves literature as the novel contribution are highlighted in theory. Furthermore, the government education policies must be reframed under the light of this research’ findings to improve students’ health.

## 1. Introduction

Mental and physical fitness is necessary for students of any class. The students are supposed to improve their health for their better performance. However, the reliability of students’ performance is based on their active learning. Mental health has a critical role in the active learning of students [1]. Conversely, college students are mentally strong because of their psychological health and better improvement as they participate in sports events. Oppositely, the students at schools are less productive than the college students due to their limited approach to learning and advanced performance [2]. Students’ health is considered a critical factor in improving their standard of living and learning performance. The situation of school students is worse in China from the perspective of their health. Almost 90% of the students from government sector schools are not entertained with sports activities [3], and this factor hinders the students’ sports performance.

Health education is not provided to school students in China to improve their health. Meanwhile, the students must improve their health standards for their productive performance. The Ministry of Education failed to develop the curriculum policies for the students to provide health education to the students in China [4]. This failure of policies making and implementation to advance students’ mental health is a significant factor in improving students’ learning. On the other hand, the students of developed countries are far better in their health than Chinese students [5]. Developed countries like Germany, Japan and Australia have introduced health education for students at the school level. This advancement in health education can improve the health status standard for Chinese students. The overall performance of school students in China is less than that of developed countries [6]. This practical problem is less discussed in the literature and this can be resolved by effective management of educational institutes [7, 8].

Scholars have neglected to pay attention to the health education of school students in China. The study Abbas, Aman [9] discussed that health education for students is necessary, but the findings of this study are based on the students at colleges and universities. However, the research by Abid, Zahid [10] highlighted that health education is a reliable source to improve students’ learning, but this research has paid little attention to the advancement of school students’ health. The studies tested the role of physical health in the students’ learning performance in Chinese institutes [11]. Hence, there is a loop in the literature as the studies have not discussed health education for the psychological well-being of the students in Chinese [12–14].

Importantly, the school students are facing mental health issues, and their performance is not improving in China [15]. Furthermore, Zhang, Yang [16] highlighted that health education policies are not implemented at the school level in China. The students’ psychological wellbeing is tested in different contexts, but not in the context of exercise. Current research is conducted to address this theoretical gap in the body of knowledge. This research goal is to test the impact of health education on the psychological well-being of school students. This research is based on stimulus–response theory as it explains the relationship between variables. In accordance, this study has introduced two moderating variables, sustainable health exercise and sports participation, to determine the impact of these factors on the relationship between health education and psychological well-being. The scope of this research is limited to the public sector schools in the Wuhan city of China. This research improves literature as the novel contribution regarding related to the role of psychological wellbeing and its antecedents such as health education, sustainable health exercise, and sports participation. Practically, this study has novelty as no study highlighted these factors for the wellbeing of students. This research is also reliable methodologically as it tested the moderating influence of two variables sustainable health exercise and sports participation on the relationship between health education and psychological wellbeing. Furthermore, the government education policies must be reframed under the light of this research’ findings to improve students’ health.

## 2. Review of literature

### 2.1 Theoretical foundations

**Fig 1:**
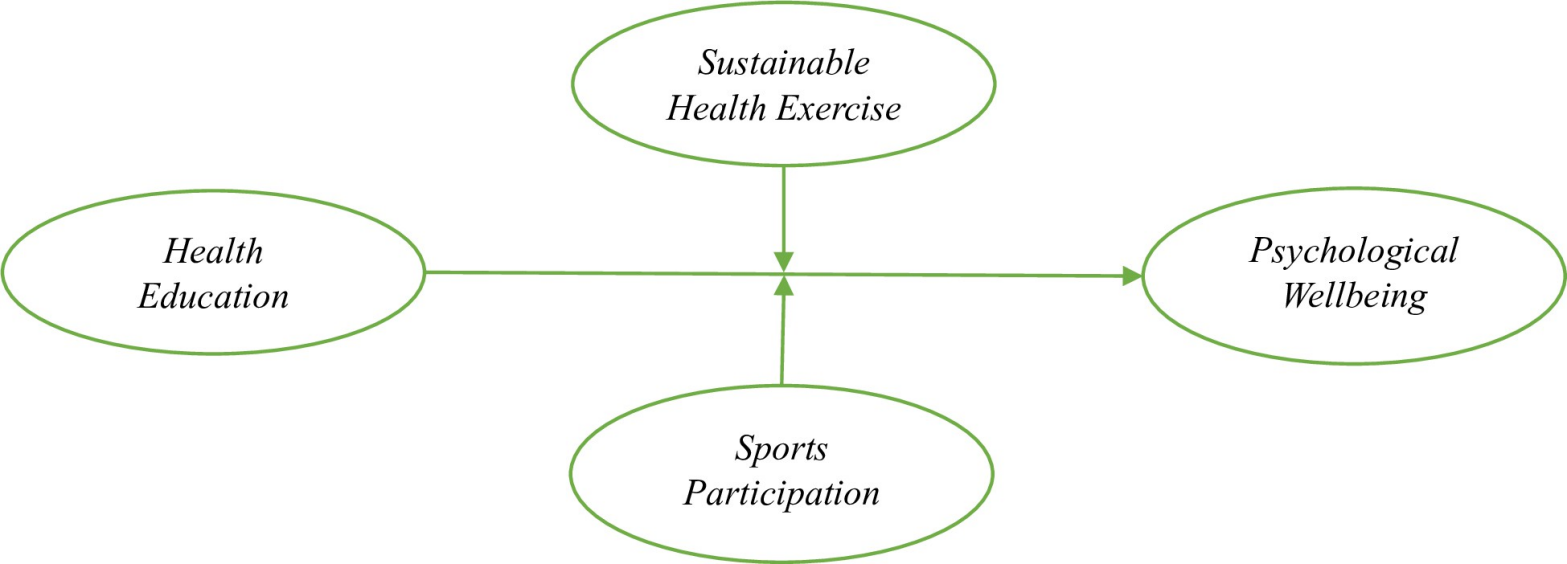
Purposed framework.

Stimulus–response theory highlights that any actions cause any results. According to this theory, the actions of individuals and groups are based on any action that stimulates these actions [17]. This theory fits in this research context because educational activities are stimuli for any results of the students. The purpose framework of this research has psychological well-being as a dependent variable that directly responds to the stimulus. Psychological wellbeing is defined as an individual’s emotional health and overall functioning [18, 19]. Accordingly, the stimulus in this study is health education, which leads the students to improve their psychological well-being [20]. As stimulus–response theory highlights, the stimulus triggers any response. In this way, the stimulus-response theory is underpinned by this research to develop the theoretical model. However, this research introduced two moderators, sustainable health education and sports participation, to determine the theoretically developed relationship between sustainable health education and psychological well-being. The proposed framework is highlighted in Fig 1.

### 2.2 Health education and psychological wellbeing

Health education is based on providing people with health-related information and awareness. The role of health education is critical in our society because people are highly motivated to improve their standard of education [21]. The advancement in public health is possible when they are appropriately educated about their health issues. When the students are motivated to improve their health, their work for health advancement is increased over time [22]. The availability of health education to students improves their behaviour and learning performance.

When the students are highly motivated to learn about their health, their awareness regarding health education improves [23]. The students must get enrollment and education-related courses. Physical and mental health is essential for students to improve their learning performance. When the students are physically appropriate, they pay deep attention to improving their health. On the other hand, when the students are not well physically, they are not supposed to improve the standard of their health [24]. Health education is critical for people, but it should be improved over time to advance the behavior and learning of people. When the students are introduced to health education, they learn how to improve their psychological and physical health. Physical health provides stamina to the students for their better learning and performance in curricular and extra-curricular activities [25, 26]. The study Rahi [27] reported that the psychological well-being of the employees has role in their psychological empowerment.

However, without the awareness of health education, students may face problems in improving the standard of their health. In developed countries, students’ health education is the primary focus of the people making education policies [28]. In this way, the students of the developed nations are far better at advancing their health. Accordingly, the students of developing nations are less productive in their health because they are not provided with healthy education appropriately. The primary role of health education is to improve the health standards of the students to make them aware of health issues [29]. The students with robust mental health and more productive performance compared to those who are not encouraged to improve their mental health. The scholars discussed health education and psychological well-being separately, but the rationale is not developed to test the direct impact of health education on students’ psychological well-being.

H1: There is an influence of health education on students’ psychological well-being.

### 2.3 Sustainable health exercise

Health exercise is necessary for students to improve their health standards. The students are motivated to improve their health and exercise to become fit physically and psychologically [30]. The impact of psychological health is necessary for the students because they must think about their tasks. However, the central role of psychological health is improved with physical exercise. The students with better psychological health are better at their performance and working as compared to the students who don’t have reliable psychological health [31]. Indeed, the health exercise units should be developed in the sports centers of the schools and colleges to educate the students to improve their health critically.

The access of people to health information and the availability of health-related behaviour improves the psychological well-being of the student. When the students are advanced to improve their health, they exercise with a proper routine [32]. The success rate of health exercise is significant for the better health performance of the students. On the other hand, those students who are not fully involved in improving their health standards face critical challenges in advancing their health. It is highlighted that students should develop a positive psychology to improve their health. Awareness of health issues and availability of the health standard is a significant factor in improving their health standard [33]. No doubt, the students involved in health exercise always keep improving their health over time. Comparatively, students who are less productive to improve their health standards face challenges.

Meanwhile, parents must motivate their children to improve their health with exercise. Routine exercise is critical for the students to improve their health because many are physical, but they can improve their psychological health with healthy exercise [34]. Moreover, the experts’ health exercise-related training leads the students toward extraordinary health exercise performance. This way, the student’s psychological well-being is improved by developing routine exercises [35]. Therefore, sustainability in health exercise is necessary to improve the health standard and behaviour of the students. The existing studies have detailed discussion regarding the relationship between health exercise for students. However, these studies have a loop in their findings as the moderating role of health exercise on the relationship between health education and students’ psychological well-being is not reported by these studies.

H2: A moderate influence of sustainable health exercise exists between health education and students’ psychological well-being.

### 2.4 Sports participation

Sports have a significant impact on human health. Participation in sports improves the health standard of the people. The participation in sports activities by the students is a significant factor in improving the standard of their health [36]. The advancement in people’s behaviour and learning provides a way for sports participation. However, when the students participate in outdoor activities and indoor sports, their health is improved. The rational well-being of the students is improved with the advancement of psychological well-being [37]. Participation in sports activities provides a way for students to improve their physical and mental health. The active participation of the students in sports activities can improve the learning performance of the students.

Meanwhile, when the students are improving their health standards, they are required to be involved in sports. Many students are involved in sports activities but must work accordingly for their health improvement [38]. Indeed, improving health is a critical task, but the students should be motivated by health education to participate in sports activities. The relationship between students and healthy activities can be vital when participating in outdoor games [39]. Indeed, the health of students, both physically and mentally, is developed with the help of sports, and it has a significant impact on the betterment of people to improve their living standards. The reliability of people’s health and their advanced performance to improve health can be a significant factor in participating in sports programs.

Motivation for students is necessary to encourage them to participate in sports [40]. When outdoor games are conducted in schools and colleges, the students should participate to improve their physical and mental performance. The reading habit of the students can be improved when they are physically and mentally stable. In this way, the role of sports performance is critical to improving the student’s understanding of advancement in their sports performance and health standard improvements [41]. The students must be motivated to participate in the sports activities because the impact of sports activities is significant on the student’s psychological health to improve their performance. The link of sports participation with students’ psychological well-being is widely discussed. However, the studies have paid less attention to measure the moderating role of sports participation on the relationship between health education and students’ psychological well-being. Hence, there is a gap in the literature.

H3: Sports participation is a moderating influence between health education and students’ psychological well-being.

## 3. Methodology

Positivism is a research paradigm or philosophical approach that prioritizes the utilization of empirical and observable data to acquire knowledge about the world [42]. The positivist paradigm is based on the conviction that the scientific method, which prioritizes objectivity, measurement, and methodical observation, is the most dependable approach to gaining knowledge [43]. This paradigm is specifically linked to the natural sciences, with the objective of identifying and elucidating regularities or patterns in the world. This research is also based on positivism.

### 3.1 Research instruments and operationalization

The quantitative data is considered in this study to test the theory with empirical evidence. The constructs of this research are operationalized according to the available research instruments. Health education is operationalized to measure the impact of health education on the students’ psychological well-being. The items considered for health education are used to collect the data from respondents while discussing health education critically. Furthermore, the study has operationalized psychological well-being to measure the data concerning health education. The research has a moderating variable sustainable health exercise, and it is operationalized to measure the impact of sustainable health exercise on students’ psychological well-being.

Similarly, another moderator, sports participation, is also operationalized to measure the impact of sports participation on psychological well-being and health exercise. This research questionnaire is divided into four sections to collect the data. Furthermore, the school administrators were requested to collect the demographic data but weren’t unwilling to provide it. Therefore, the section on demographic data was eliminated from the research questionnaire. The scale items for health education are adapted from Lai, Wu [44]. Furthermore, the scale items for psychological wellbeing are adapted from Spreitzer [45]. Thirdly, the scale items for sustainable health exercise are adapted from Kamrani, Sani [46]. Finally, the scale items for sports participation are adapted from Khan, Jamil [47].

### 3.2 Data collection and sampling technique

The population of this research were the students of government and private sector schools in China. However, the research frame was limited to the population of Wuhan city because it is feasible to collect data. Furthermore, the students of 10^th^ grade are mature and participate in health education classes selected for data collection. Two clusters of 36 schools were made, each containing 18 schools. These clusters were made based on the public and private sector schools. The population of the study were informed about the research questionnaire. The students were highly motivated to provide data for this research.

Furthermore, the data is collected randomly from the students in 10^th^ grade. Six hundred printed questionnaires were distributed to collect the data without any bias. However, 567 questionnaires were returned, but 18 were not filled in correctly. Therefore, 549 is considered the sample size of this research.

### 3.3 Statistical tool

The collected data of this research is analyzed with the findings of Smart PLS 4. The convergent validity test and discriminant validity findings are determined with the findings of the measurement model assessment. However, structural model assessment findings are used to test path findings. Furthermore, predictive relevance was also tested in the concluding part of the findings section.

## 4. Data analysis and findings

The normality of distribution is checked at the initial stage of data analysis. The findings of skewness and kurtosis are used to test data normality. The findings of skewness and kurtosis between -2 and +2 [48] are considered appropriate for significant normality of data. Furthermore, the missing values were tested, and there was no missing value in the data. The findings of the normality of distribution are reported in Table 1.

**Table 1:** Normality of distribution.

No.	Items	Missing	Mean	Median	Min	Max	Standard Deviation	Excess Kurtosis	Skewness
1	PW1	0	3.244	3	1	7	1.523	-0.491	0.099
2	PW2	0	3.262	3	1	7	1.813	-0.584	0.438
3	PW3	0	3.529	3	1	7	1.882	-0.811	0.298
4	PW4	0	3.502	3	1	7	1.916	-0.814	0.37
5	PW5	0	3.552	3	1	7	1.739	-0.492	0.304
6	PW6	0	3.511	4	1	7	1.829	-0.726	0.236
7	HE1	0	3.502	4	1	7	1.839	-0.869	0.159
8	HE2	0	3.679	4	1	7	1.864	-0.776	0.189
9	HE3	0	3.701	3	1	7	1.872	-0.76	0.319
10	HE4	0	3.692	3	1	7	1.944	-0.807	0.343
11	HE5	0	3.584	3	1	7	1.897	-0.732	0.38
12	SHE1	0	3.584	3	1	7	1.852	-0.626	0.362
13	SHE2	0	3.602	3	1	7	1.913	-0.799	0.335
14	SHE3	0	3.475	3	1	7	1.784	-0.459	0.45
15	SHE4	0	3.52	4	1	7	1.921	-0.914	0.22
16	SHE5	0	3.462	3	1	7	1.826	-0.643	0.311
17	SP1	0	3.633	3	1	7	1.779	-0.599	0.284
18	SP2	0	3.077	3	1	7	1.486	-0.128	0.593
19	SP3	0	3.19	3	1	7	1.504	0.435	0.885
20	SP4	0	3.226	3	1	7	1.453	0.753	0.91
21	SP5	0	3.172	3	1	7	1.451	0.458	0.779

**Fig 2:**
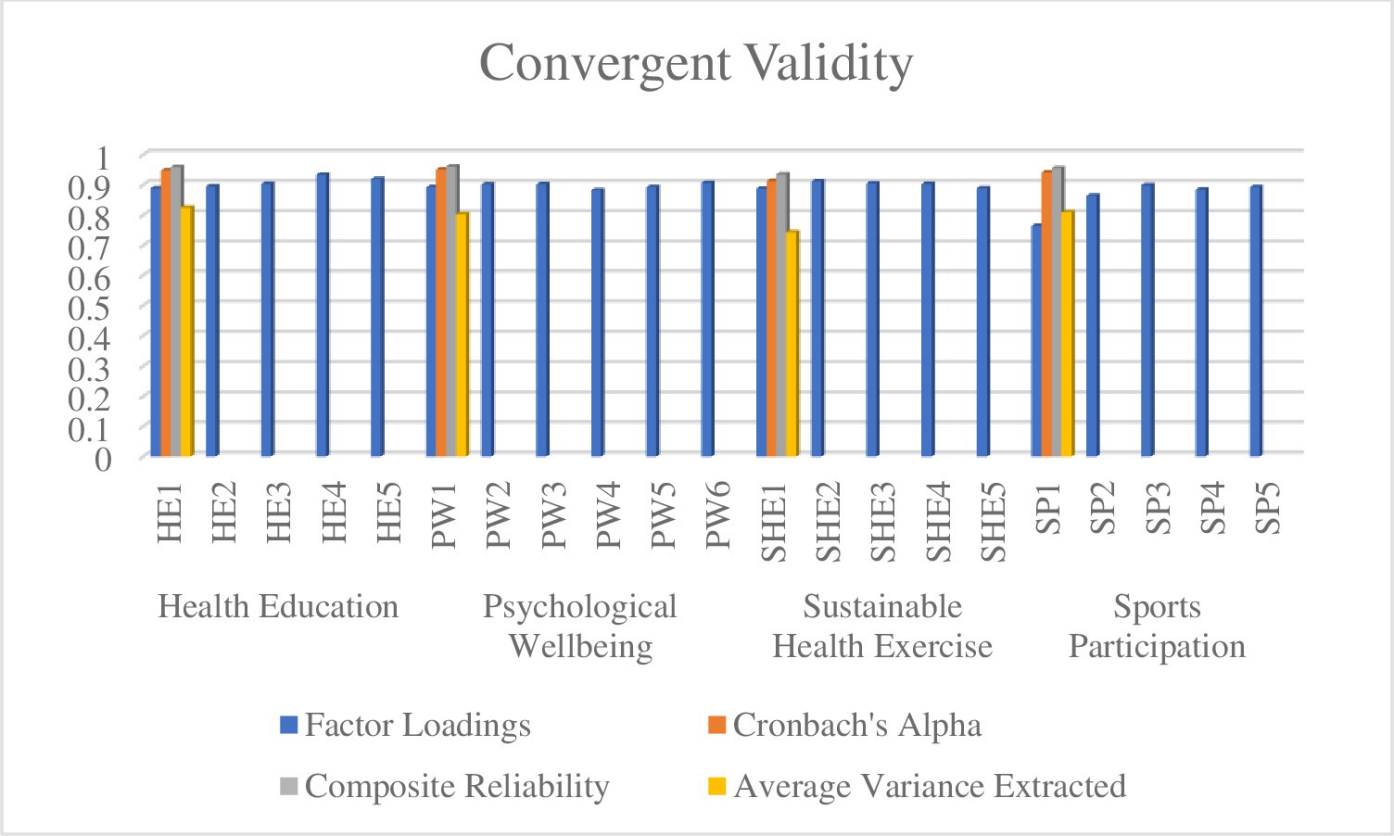
Convergent validity.

The findings of convergent validity are tested to check the validity of internal items and internal consistency between the research data. The findings of factor loadings > 0.60 [49] are considered to test the reliability of individual items. In this way, the Cronbach alpha and composite reliability results are checked to determine the internal consistency between the constructs. The findings of composite reliability > 0.70 and Cronbach alpha > 0.70 [50] are considered significant for research data. Therefore, the convergent validity of research data is also achieved. The findings of average variance extraction are tested to determine the variance of items loaded on constructs. The results highlighted that all items loaded on constructs were more than 50% [51]. The findings of convergent validity and reported in Table 2 and Fig 2.

**Table 2:** Convergent validity.

Variables	Items	Factor Loadings	Cronbach’s Alpha	Composite Reliability	Average Variance Extracted
Health Education	HE1	0.889	0.947	0.959	0.825
	HE2	0.895			
	HE3	0.903			
	HE4	0.934			
	HE5	0.919			
Psychological Wellbeing	PW1	0.892	0.951	0.961	0.803
	PW2	0.901			
	PW3	0.902			
	PW4	0.882			
	PW5	0.892			
	PW6	0.907			
Sustainable Health Exercise	SHE1	0.888	0.913	0.935	0.743
	SHE2	0.911			
	SHE3	0.905			
	SHE4	0.903			
	SHE5	0.889			
Sports Participation	SP1	0.764	0.941	0.955	0.809
	SP2	0.864			
	SP3	0.899			
	SP4	0.884			
	SP5	0.892			

The findings of discriminant validity are checked to determine the multicollinearity issues in the research data. The findings of Heteritrait-Monotrait (HTMT) and cross-loadings are tested to determine the discriminant validity. HTMT is considered significant in any research when the findings of the HTMT matrix are below 0.90 [52]. The reported results in Table 3 highlighted that the research achieved significant discriminant validity. Accordingly, the cross-loadings are significant when the items loaded on one construct are higher in loading as compared to the other constructs that are correlated [53]. The results of cross-loadings are highlighted in Table 4 and are significant.

**Table 3:** HTMT.

Variables	Health Education	Psychological Wellbeing	Sports Participation	Sustainable Health Exercise
Health Education				
Psychological Wellbeing	0.791			
Sports Participation	0.787	0.825		
Sustainable Health Exercise	0.698	0.777	0.785	

**Table 4:** Cross loadings.

Variables	Items	Health Education	Psychological Wellbeing	Sports Participation	Sustainable Health Exercise
Health Education	HE1	0.889	0.864	0.692	0.816
	HE2	0.895	0.847	0.66	0.851
	HE3	0.903	0.847	0.696	0.885
	HE4	0.934	0.867	0.677	0.871
	HE5	0.919	0.844	0.676	0.856
Psychological Wellbeing	PW1	0.827	0.892	0.748	0.835
	PW2	0.833	0.901	0.661	0.821
	PW3	0.839	0.902	0.663	0.835
	PW4	0.84	0.882	0.72	0.821
	PW5	0.849	0.892	0.726	0.834
	PW6	0.868	0.907	0.701	0.822
Sustainable Health Exercise	SHE1	0.811	0.82	0.675	0.888
	SHE2	0.851	0.827	0.638	0.911
	SHE3	0.835	0.818	0.646	0.905
	SHE4	0.895	0.864	0.688	0.903
	SHE5	0.843	0.825	0.71	0.889
Sports Participation	SP1	0.808	0.813	0.764	0.832
	SP2	0.589	0.616	0.864	0.573
	SP3	0.61	0.652	0.899	0.609
	SP4	0.567	0.614	0.884	0.562
	SP5	0.578	0.62	0.892	0.559

**Fig 3:**
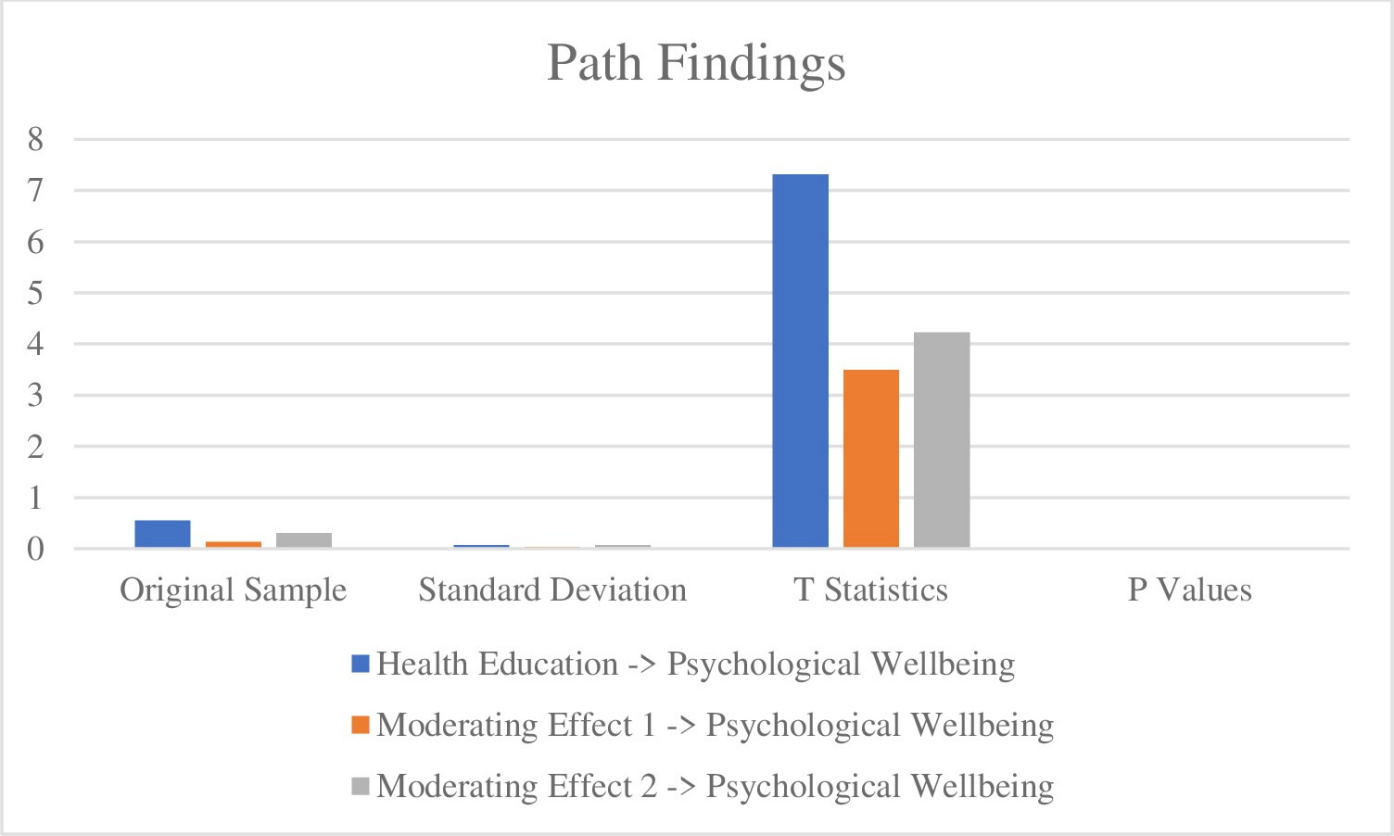
Path findings.

The findings of t-statistics are considered to collect the data. The results are taken with the findings of the structural model assessment. The t values of more than 1.96 are acceptable for significant results [54]. The findings of the first hypothesis confirmed that health education significantly impacts the students’ psychological well-being. Furthermore, the findings of the second hypothesis reported that sustainable health exercise moderates the relationship between health education and the student’s psychological well-being. Accordingly, the findings of the third hypothesis reported that sports participation moderates the relationship between health education and the student’s psychological well-being. The results are reported in Table 5 and Fig 3.

**Table 5:** Path findings.

No	Paths	Original Sample	Standard Deviation	T Statistics	P Values	Results
1	Health Education -> Psychological Wellbeing	0.552	0.075	7.321	0	Accepted
2	Moderating Effect 1 -> Psychological Wellbeing	0.136	0.039	3.499	0.001	Accepted
3	Moderating Effect 2 -> Psychological Wellbeing	0.308	0.073	4.231	0	Accepted

**Fig 4:**
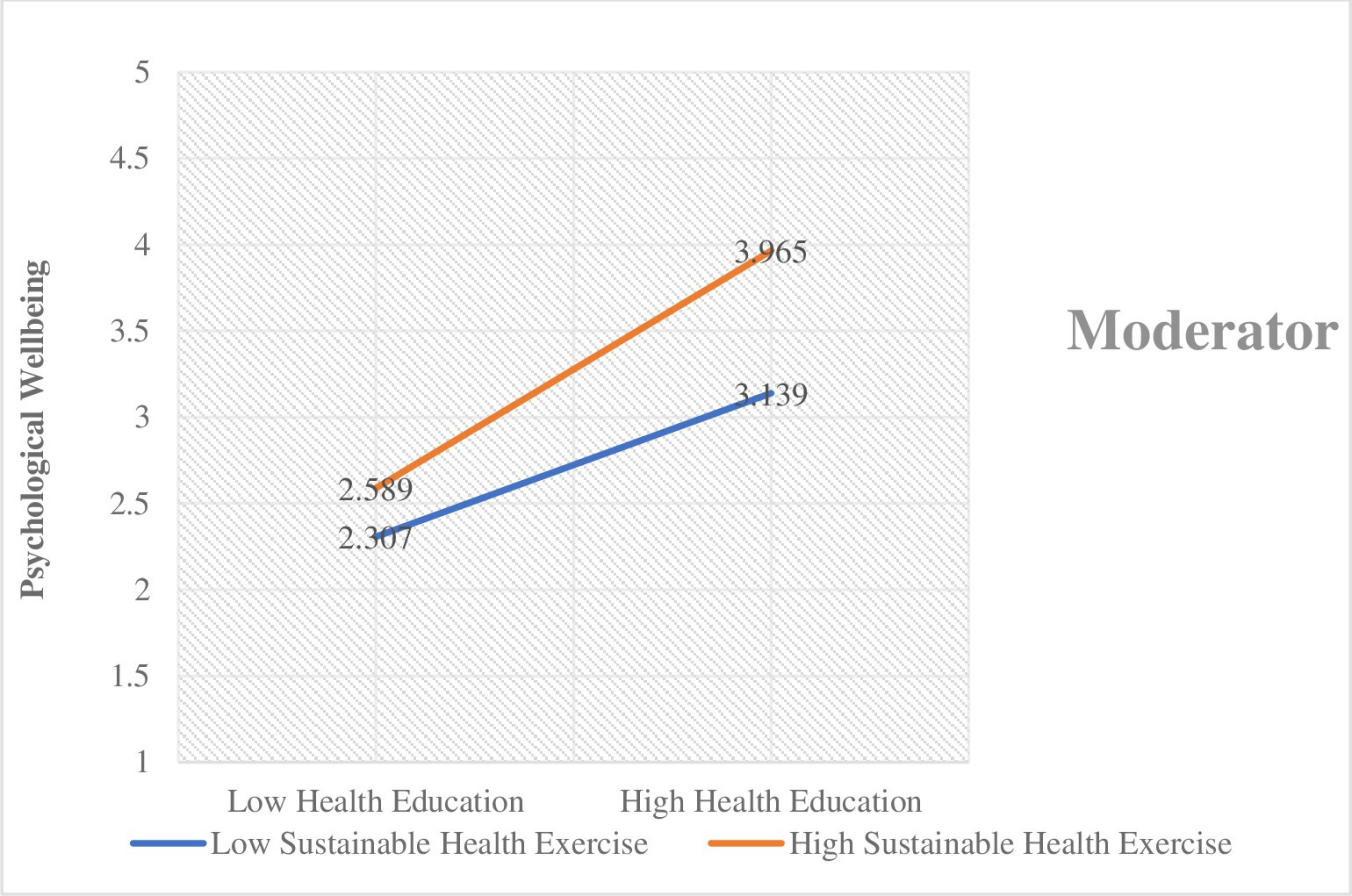
Moderating effect 1.

The findings demonstrated that the increase in sustainable health exercise increases the effect of health education on the students’ psychological well-being. On the other hand, the decrease in sustainable health exercise decreases the effect of health education on the students’ psychological well-being. This relationship is graphically presented in Fig 4.

**Fig 5:**
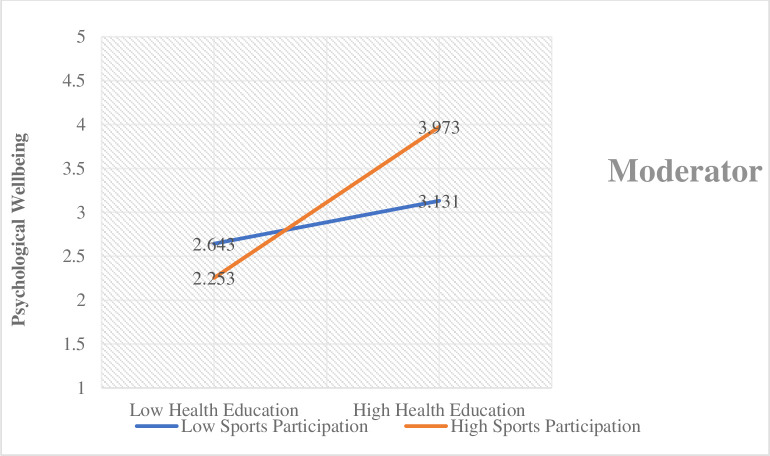
Moderating effect 2.

In accordance, the findings demonstrated that the increase in sports participation increases the effect of health education on the students’ psychological well-being. On the other hand, the decrease in sports participation decreases health education’s effect on the students’ psychological well-being. This relationship is graphically presented in Fig 5.

The findings of predictive relevance are used to determine the relevance of the model and disclose the relationship between constructs. The findings of predictive relevance more than 0 are considered significant [55]. The model of this research has 72% predictive relevance of health education on students’ psychological well-being. The reported results in Table 6 confirmed that this study has appropriate predictive relevance in the model.

**Table 6:** Predictive relevance.

Variables	SSO	SSE	Q^2^ (= 1-SSE/SSO)
Health Education	1105	1105	
Psychological Wellbeing	1326	369.204	0.722
Sports Participation	1105	1105	
Sustainable Health Exercise	1105	1105	

## 5. Discussion

The objective of this research is achieved with the findings of its hypotheses. The direct and moderating hypotheses are empirically supported. To begin with, the findings of the first hypothesis showed that the effect of health education is significant on the students’ psychological well-being. This relationship was theoretically developed and empirically justified by this research. Although this relationship is newly developed in the literature, findings of existing studies empirically and theoretically support this relationship. According to Alotaibi, Fox [33], students may encounter difficulties raising their level of health if they are not aware of health education. The primary concern of those who create educational policies in industrialized nations is the health education of kids.

According to Guszkowska and Dąbrowska-Zimakowska [30], the pupils in wealthy countries are far better at advancing their health in this way. As a result of inadequate health education, students in underdeveloped countries are less productive in terms of their health. According to Grande, Berdida [36], the main goal of health education is to raise students’ health standards and increase their awareness of health issues. Compared to pupils who are not pushed to enhance their mental health, those with significant mental health perform better overall. According to Dragun, Veček [32], students’ physical and mental well-being is crucial for enhancing their academic success. When students are in good physical condition, they focus intensely on raising their level of health. On the other side, pupils are not supposed to raise their level of health when they are not physically well. According to Schmidt, Reinboth [35], although health education plays a vital part in people’s lives, it should be enhanced throughout time to advance human behaviour and knowledge. Students exposed to health education learn how to enhance their physical and mental well-being.

According to Hartman, Barcelona [37], the pupils’ physical health gives them the stamina they need for more significant learning and performance in both academic and extracurricular activities. Health education is founded on ideas that aim to teach and educate individuals about their health. According to Pastor, Cervelló [34], due to the high level of motivation for education improvement in modern society, the importance of health education cannot be overstated. Public health can advance when people are informed enough about their health problems. When students are inspired to take better care of themselves, their efforts to progress their health grow. According to Leavell, Leiferman [31], the pupils’ behaviour and academic performance are improved by providing health education. Students’ awareness of health education increases when they are incredibly motivated to learn about it. Getting enrolled in and taking education-related courses is essential for students.

In accordance, the findings of the second hypothesis showed that the effect of health education is significant on the students’ psychological well-being with the moderating role of sustainable health exercise. Also, this relationship was theoretically developed and empirically justified by this research. Even though this relationship is newly developed in the literature, the findings of existing studies empirically and theoretically support this relationship. According to Neufeld and Malin [40], there is little doubt that kids who participate in healthy activity consistently improve their health over time. In contrast, students who are less successful in improving their health standards struggle. In the meanwhile, parents must encourage their kids to exercise to raise their level of health.

According to Bean, McFadden [38], since many students struggle physically, regular exercise is essential to enhance their health. However, they can also benefit psychologically from regular exercise. Additionally, the pupils’ extraordinary health exercise achievement results from the specialists’ training in the field. According to Jusienė, Breidokienė [56], students who acquire regular exercise habits have an improvement in their psychological health. The prevalence of health-related behaviours and people’s access to health information boosts the student’s psychological well-being. According to Agrawal and Krishna [41], when students are ready to advance in improving their health, they engage in regular exercise. The success rate of health exercises significantly impacts the pupils’ improved health performance. On the other hand, students who are not totally committed to raising their health level may find significant learning obstacles.

According to Idris, Zulkipli [57], it is emphasized that the pupils should cultivate positive psychology to raise their level of health. The availability of the health standard and understanding of health issues are important factors in raising their health standard. Students must engage in regular physical activity to raise their level of health. According to Calderon Jr, Pupanead [39], the children are inspired to exercise and improve their health to become mentally and physically fit. Students must consider the effects of psychological wellness because they have work to do. However, exercise plays a significant part in improving psychological health, which plays a critical impact. According to González-Hernández, Gómez-López [58], compared to pupils who don’t have consistent psychological health, students with more excellent psychological health perform and work better. To teach students how to improve their health significantly, health exercise units should be built in the sports facilities of schools and colleges.

Lastly, the findings of the second hypothesis showed that the effect of health education is significant on students’ psychological well-being with the moderating role of sports participation. Similarly, this relationship was theoretically developed and empirically justified by this research. Although this relationship is newly developed in the literature, the findings of existing studies empirically and theoretically support this relationship. According to Qin, Song [23], a person’s ability to maintain or enhance their health can significantly influence participation in sports programs. Students must be motivated to be encouraged to participate in sports. According to Rutkowska, Liska [28], learners should participate in outdoor competitions at their schools and universities to improve their physical and mental performance. When pupils have good physical and mental health, their reading habits can be enhanced. Learners must participate in athletics while working to improve their health.

According to Zhang, Mavoa [29], although many students participate in sports, they must improve their health. Although it is essential to improve one’s health, pupils should be encouraged to participate in athletic activities through health education. When pupils play outdoor games, there can be a strong connection between their participation and healthy behaviours. According to Belcher, Zink [24], sports assist pupils in building their physical and mental health, significantly impacting how individuals are treated and their living standards are raised. Sports significantly impact human health. Sports participation raises peoples’ standards of health. The grade of the pupils’ health is improved significantly by their participation in sporting activities. Many students were effected to get online education during covid-19 [59].

According to Budzynski-Seymour, Conway [60], the development in human behaviour and learning opens opportunities for engagement in sports. However, the pupils’ health improves when engaging in indoor and outdoor sports. The improvement of psychological wellness benefits students’ cognitive well-being. According to Cilar, Štiglic [22], sports engagement allows pupils to advance in enhancing their psychological and mental well-being. Their active engagement in sporting events can enhance students’ learning performance. In this sense, a student’s comprehension of the function of sports performance in improving their sports performance and health standards is crucial. According to Pourranjbar, Khodadadi [21], the pupils must be inspired to participate in sports because they significantly impact their psychological well-being and help them perform better. Meanwhile, Saddique, Safdar [61] reported that the awareness level of students has significant impact on their health management.

## 6. Theoretical, practical implications, contribution, conclusion and future directions

The current research is concluded with strong theoretical justifications in the literature. It has empirically tested the relationship that was developed theoretically. The findings of this research highlighted that health education is a critical factor in advancing students’ psychological well-being. By its operationalization, the study demonstrated that health education teaching students improve their health awareness, directly impacting their learning performance. The study also highlighted that health education teaching to students improves their behaviour towards developing education to improve their psychological health.

The study also asserted that the student’s health education teaching for students is necessary as it motivates them to be healthy and fit all the time. Furthermore, the study also reported that the students feel comfortable when they believe that health education is a reliable way to improve their standard of living with appropriate performance. The findings of this research improve the literature on health education of students. Hence, a significant loop in the body of knowledge is closed by the findings of this research. The research also introduced two moderating variables sustainable health exercise and sports participation. The findings of this study highlighted that these two moderating variables are positively improving the relationship between health education and psychological wellbeing of the students.

This research has some practical implications for educational institutes, particularly schools in China. The study encourages the administration of schools to improve health education teaching to the student to advance their learning performance. Furthermore, health education is critical for the students to improve their performance; with the advancement of this education, students become more efficient towards their learning and performance. In accordance, the students are required to have proper health education while in school because awareness of their health is a significant factor in improving their learning performance.

Meanwhile, the students must improve their sports participation because participation in sports is a significant factor in improving their psychological health. Participation in sports makes the students efficient regarding their performance, and the student’s psychological well-being is improved. At the same time, the students must have sustainability in their physical exercise because the role of physical exercise is critical to learning. The research asserted that the government should improve health education teaching at the school level to improve student’s mental health and get better productivity.

### 6.1 Research contribution and conclusion

This research is based on quantitative method to test the data. The survey-based questionnaire was administrated to collect the data for this research. Furthermore, this research is based on the underpinning of stimulus–response theory which is considered for the first time this area of research. This theory is empirically supported by the findings of this study. Therefore, the finding of this study is useful for the practitioners to develop the health education for students considering it as a stimulus for the improvement of students’ psychological wellbeing.

The results indicated that health education had a substantial impact on the psychological well-being of school kids in China. The study highlighted the crucial significance of sustainable health exercise and sports engagement as a moderator, which has a favorable impact on the link between health education and psychological well-being. This research enhances the existing body of literature by emphasizing the innovative contributions in theory. Moreover, the government should revise its education policies considering these research findings to enhance students’ health education and promote their overall well-being and academic achievement.

### 6.2 Limitations and future directions

There is a need for future studies in this area. Firstly, this study has limitation because data is collected from China only which can’t predict the generalization of findings. Therefore, future studies are required to collect data from the students in Nepal and Bangladesh schools to develop a comparative analysis between the research findings. This study scope was limited to test the impact of health education on psychological wellbeing of the students. However, future studies are required to determine the role of health awareness seminars in the health education of students. In this way, future studies’ findings would contribute significantly to the body of knowledge and provide ground for significant implications.

## Supporting information

S1 Data (XLSX)
